# Factors Influencing Moroccans’ Intention to Get the COVID-19 Vaccine: Results From a Cross-Sectional Study

**DOI:** 10.7759/cureus.73051

**Published:** 2024-11-05

**Authors:** Fatima zahra Chellat, Nassiba Bahra, Zakia Marso, Marwa Elbaldi, Soumaya Benmaamar, Nabil Tachfouti, Nada Otmani, Mohamed Berraho, Karima El Rhazi

**Affiliations:** 1 Laboratory of Epidemiology, Clinical Research and Community Health, Faculty of Medicine and Pharmacy, Sidi Mohamed Ben Abdellah University, Fez, MAR; 2 Laboratory of Epidemiology and Research in Health Sciences, Faculty of Medicine and Pharmacy, Sidi Mohamed Ben Abdellah University, Fez, MAR

**Keywords:** coronavirus disease 2019 (covid-19), covid-19, covid-19 outbreak, covid-19 pandemic, covid-19 vaccination hesitancy, intention, morocco, vaccination, vaccine

## Abstract

Background

During the COVID-19 pandemic, the introduction of the vaccine represented a light of hope. With the vaccine now accessible globally, the focus shifts to encouraging community vaccination to attain herd immunity. This study describes the factors that affect individuals’ willingness to get vaccinated in Morocco.

Methods

From April 9, 2022, to April 27, 2023, a cross-sectional survey was conducted among Moroccan individuals aged 18 and older at primary healthcare centers. The survey was administered through anonymous questionnaires and explored various factors that influence people’s intentions regarding COVID-19 vaccination. These factors included sociodemographic and clinical characteristics, perceptions of COVID-19 risk, knowledge about vaccines, and concerns about vaccine safety and efficacy. Chi-square tests or Student’s t-tests were used to describe these variables, and significant determinants of vaccine intention were identified using a logistic regression model.

Results

Among the 453 participants, 77.5% were women. The mean age was 41.4±16.6 years. Among the surveyed group, 51.2% indicated that they did not intend to get vaccinated against COVID-19. Several factors were significantly associated with the willingness to be vaccinated (p<0.05), including having no income (adjusted OR (AOR): 2.7, 95% confidence interval (CI): 1.9-5.1, p=0.003), believing that COVID-19 is a serious infection (AOR: 13.1, 95% CI: 7.6-22.3, p<0.001), not having had a COVID-19 infection (AOR: 3.3, 95% CI: 1.7-6.4, p=0.001), having concerns about vaccine side effects (AOR: 8.6, 95% CI: 4.4-16.9, p<0.001), and believing in the benefit of booster doses (AOR: 5.5, 95% CI: 2.4-12.6, p<0.001).

Conclusion

The low vaccination intent observed in this study presents a major challenge for controlling COVID-19 and potentially other infectious diseases because the determinants of vaccine intent are likely similar. This finding underscores the urgent need for reliable, authoritative information on the risks of disease, the efficacy of the vaccine, and the benefits of vaccination. Additionally, assessing the commitment to completing full vaccination schedules is crucial to enhancing population-wide vaccine uptake.

## Introduction

The rapid spread of COVID-19 worldwide prompted the World Health Organization (WHO) to declare it a pandemic and a Public Health Emergency of International Concern (PHEIC) [1]. Various strategies were deployed in response to this crisis, including testing, treatment, and established preventive measures. Among these strategies, COVID-19 vaccination emerged as the most effective and cost-efficient preventive measure by significantly mitigating the pandemic’s impact and providing protection against the virus [2]. While traditional vaccine development typically occurs over a decade on average, the development of COVID-19 vaccines occurred at an unprecedented pace to achieve herd immunity, which normally necessitates vaccination rates of approximately 85% to prevent a severe increase in infections [3]. Consequently, world leaders and public health authorities vigorously endeavored to secure an ample supply of COVID-19 vaccines and to devise effective deployment strategies to achieve herd immunity [4]. Despite this remarkable progress, convincing the population to embrace COVID-19 vaccination remained a significant challenge, as emphasized by the WHO in 2021 [5]. Additionally, while high-income countries achieved 60%-80% vaccination rates with at least one dose by mid-October 2021, developing nations faced much lower rates, such as 52% in India and less than 10% across much of Africa [6]. Globally, numerous studies have investigated COVID-19 vaccine uptake intentions, and these studies have revealed that the factors that influence vaccination intentions range from personal sociodemographic attributes and individual convictions to broader external or organizational influences [7].

In Morocco, a similar trend persists despite the launch of a vaccination campaign on January 28, 2021, that offered free access to the COVID-19 vaccine for the entire population. The analysis of vaccination coverage reveals persistently low rates, particularly concerning the administration of second and third doses, which presents a significant obstacle to reaching national herd immunity goals. This deficiency in coverage persisted even a year after the campaign’s initiation [8].

Confronted with this reality and the paucity of comprehensive data regarding the critical factors influencing the Moroccan population’s vaccination intentions, conducting a thorough examination of these determinants is imperative, particularly among those hesitant to be vaccinated. This hesitancy, as defined by the WHO, entails a delay in acceptance or outright refusal of vaccination despite the availability of vaccination services [5].

This study offers an in-depth exploration of the different factors that influence the intention to be vaccinated among the population hesitant about the COVID-19 vaccine. Specifically, the study highlights the barriers and concerns contributing to decreased vaccination intent, which provides valuable information to policymakers. Additionally, the study identifies key factors that necessitate action to enhance population adherence to mass vaccination campaigns.

## Materials and methods

Study design and population

The study population comprised adults aged 18 and older who were hesitant to receive the freely available COVID-19 vaccine. Participants were recruited from all the primary healthcare centers in Fez between April 9, 2022, and April 27, 2023. A sample size of 362 individuals was calculated using the formula n=P(1-P)Z^2α/i^2, with a 95% confidence level (Z=1.96), a prevalence rate (P) of 62% based on a previous Arab world study [9], and a precision (i) of 5%.

Data collection

Data was collected through face-to-face interviews with consenting participants using a questionnaire that featured 17 closed-ended questions. The questionnaire was developed by the epidemiology, clinical research, and community health laboratory teams based on thorough literature reviews to ensure its relevance. Specifically tailored to this study’s context [3,10-13], the questionnaire underwent a pilot feasibility study with volunteers from health centers. This pilot helped identify and address potential misunderstandings and difficulties, which led to revisions before the final implementation.

Study variables

Predictors

The factors examined in this study were organized into three categories: (1) sociodemographic and clinical factors, (2) perceived risk of contracting COVID-19, and (3) factors related to knowledge, concerns, and confidence regarding COVID-19 vaccination [3,10-13].

The sociodemographic and clinical factors included variables such as age, gender (female or male), marital status (married or unmarried), education level (illiterate or literate), monthly household income (with or without income), employment status (active or inactive), and whether the individual was a health professional (Yes/No). Clinical factors also considered personal medical history, specifically the presence of chronic diseases such as obesity, diabetes, cardiovascular diseases, cancer, and other pre-existing chronic conditions.

In terms of the perceived risk of contracting COVID-19, the participants were asked about their beliefs regarding the severity of a COVID-19 infection, their past infections, experiences of COVID-19 within their household, and any encounters with COVID-19-related deaths. The questions for this section included, “In your opinion, is the COVID-19 infection a serious infection?” (Yes/No), “Have you already been infected with COVID-19?” (Yes/No), “Has one of the members of your social circle experienced a COVID-19 infection?” (Yes/No), “Have you experienced the death of one of the members of your social circle following a COVID-19 infection?” (Yes/No).

The questions related to knowledge, concerns, and confidence about the COVID-19 vaccine assessed the participants’ self-perceived knowledge about the vaccine, their beliefs about its effectiveness and booster doses, their understanding of the potential adverse effects, and their overall confidence in the vaccine. For this section, the questions included, “Are you sufficiently informed about the COVID-19 vaccines available and their usefulness?” (Yes/No), “Does the COVID-19 vaccine have any serious adverse effects?” (Yes/No), “Are you confident about the COVID-19 vaccine’s efficacy?” (Yes/No), and “Do you believe in the benefit of all booster doses of the COVID-19 vaccine to ensure the effectiveness of the vaccine?” (Yes/No).

To understand participants’ willingness to get vaccinated in the future, they were directly asked, “Would you get the COVID-19 vaccine in the future?” The responses were captured using a Likert scale with options ranging from “Definitely will” and “Probably will” to “Probably won’t” and “Definitely won’t” [14].

Outcome

The main outcome of interest was COVID-19 vaccine intention. The responses of “Probably will” and “Definitely will” were both considered indicative of a positive attitude and a high likelihood of vaccination [15]. Therefore, these responses were grouped as “Yes” (likely to get vaccinated). In contrast, those who responded “Probably not” or “Definitely not” were categorized as “No” (unlikely to get vaccinated).

Ethical issues

The information was collected anonymously, and the participants voluntarily agreed to participate in the study by signing a consent form. Furthermore, the participants received no incentives for their involvement. Throughout the process of data collection and analysis, strict measures were implemented to ensure the confidentiality of the data. The Ethics Committee of Fez University Hospital granted approval number 01/22 for this study.

Statistical analysis

Data entry was completed using Microsoft Excel 2013 (Microsoft Corp., Redmond, WA), and the analysis was conducted using IBM SPSS Statistics software version 26 (IBM Corp., Armonk, NY). A descriptive analysis was employed, utilizing percentages to illustrate qualitative variables and means with standard deviations (SD) for quantitative variables.

During the analysis, the study population was categorized into two groups: (1) individuals who are hesitant but determined to vaccinate themselves against COVID-19 in the future and (2) participants who are hesitant and not willing to accept a COVID-19 vaccine in the future. A bivariate analysis was conducted using either a chi-square test or Student’s t-test. This step was followed by a multivariate analysis using logistic regression to explore the relationship while controlling for potential confounders. Variables with a significance level of p<0.2 were included in the model, with the final significance threshold set at p<0.05.

## Results

Description of sociodemographic and clinical factors

Among the 453 volunteers participating in the study, with a mean age of 41.4±16.6 years, 351 (77.5%) were women. Of the participants, 22.5% were married. Regarding education level and career pathway, 334 (73.7%) were literate, and 186 (41.1%) were professionally active, with 99 (21.9%) of them being health workers. Among those who disclosed their income (445 participants), 86 (19%) had an income. Additionally, 167 (36.9%) participants had a history of chronic illness (Table 1).

**Table 1:** Sociodemographic and clinical characteristics and COVID-19 vaccine intention N: number, M (±SD): mean ± standard deviation *Student’s t-test was used to compare continuous variables. **The chi-square test was used to compare categorical variables. A p-value of <0.05 was considered significant.

Variables	N (%) or M (±SD) (N=453)	Vaccine intention		Test value	p-value
		Yes (n (%) or M (±SD)) (N=221)	No (n (%) or M (±SD)) (N=232)		
Age (mean ± SD)	41.4±16.6	42.2±16.6	40.1±16.6	0.807*	0.370
Gender (N=453)					
Male	102 (22.5)	48 (47.1)	54 (52.9)	0.157**	0.692
Female	351 (77.5)	173 (49.3)	178 (50.7)		
Marital status (N=453)					
Married	251 (22.5)	120 (47.8)	131 (52.2)	0.215**	0.643
Unmarried	202 (77.5)	101 (50)	101 (50)		
Level of education (N=453)					
Illiterate	119 (26.3)	61 (51.3)	58 (48.7)	0.396**	0.529
Literate	334 (73.7)	160 (47.9)	174 (52.1)		
Income (N=445)					
Without income	359 (79.2)	187 (52.1)	172 (47.9)	7.145**	0.008
With income	86 (19)	31 (36)	55 (64)		
Profession (N=453)					
Employed	186 (41.1)	83 (44.6)	103 (55.4)	2.188**	0.139
Unemployed	267 (85.9)	138 (51.7)	129 (48.3)		
Healthcare worker (N=453)					
Yes	99 (21.9)	48 (48.5)	51 (51.5)	0.005**	0.946
No	354 (78.1)	173 (48.9)	181 (51.1)		
History of chronic illness (N=453)					
Yes	167 (36.9)	83 (49.7)	84 (50.3)	0.089**	0.766
No	286 (63.1)	138 (48.3)	148 (51.7)		

Description of the perceived risk of contracting COVID-19

Regarding the perception of the risk associated with COVID-19 infection, 251 (55.4%) participants acknowledged its severity. Furthermore, a substantial majority, comprising 83 (18.3%), had personally experienced COVID-19, while 370 (81.7%) had not. Among the participants, 330 (72.8%) lived with a friend or family member who had been diagnosed with COVID-19. Moreover, 149 (32.9%) participants had lost at least one relative due to complications from COVID-19 (Table 2).

**Table 2:** Description of the associated factors with COVID-19 vaccine intention N: number, M (±SD): mean ± standard deviation **The chi-square test was used to compare categorical variables. A p-value of <0.05 was considered significant.

Variables	N (%) or M (±SD) (N=453)	Vaccine intention		Test value	p-value
		Yes (N (%)) (N=221)	No (N (%)) (N=232)		
Perceived risk of contracting COVID-19					
In your opinion, is the COVID-19 infection a serious infection?					
Yes	251 (55.4)	181 (72.1)	70 (27.9)	122.576**	<0.001
No	202 (44.6)	40 (19.8)	162 (80.2)		
Have you already been infected with COVID-19?					
Yes	83 (18.3)	27 (32.5)	56 (67.5)	10.747**	0.001
No	370 (81.7)	194 (52.4)	176 (47.6)		
Has one of the members of your social circle experienced a COVID-19 infection?					
Yes	330 (72.9)	171 (51.8)	159 (48.2)	4.473**	0.034
No	123 (27.1)	50 (40.7)	73 (59.3)		
Have you experienced the death of one of the members of your social circle following a COVID-19 infection?					
Yes	149 (32.9)	95 (63.8)	54 (36.2)	19.921**	<0.001
No	304 (67.1)	126 (41.4)	178 (58.6)		
Factors related to knowledge, concerns, and confidence regarding COVID-19 vaccination					
Are you sufficiently informed about the COVID-19 vaccines available and their usefulness?					
Yes	31 (6.8)	18 (58.1)	13 (41.9)	1.147**	0.284
No	422 (93.2)	203 (48.1)	219 (51.9)		
Does the COVID-19 vaccine have any serious adverse effects?					
Yes	109 (24.1)	17 (15.6)	92 (84.4)	63.283**	<0.001
No	344 (75.9)	204 (59.3)	140 (40.7)		
Are you confident about the COVID-19 vaccine’s efficacy?					
Yes	107 (23.6)	85 (79.4)	22 (20.6)	52.684**	<0.001
No	346 (76.7)	136 (39.3)	210 (60.7)		
Do you believe in the benefit of all booster doses of the COVID-19 vaccine to ensure the effectiveness of the vaccine?					
Yes	78 (17.2)	66 (84.6)	12 (15.4)	48.413**	<0.001
No	375 (82.8)	155 (41.3)	220 (58.7)		

Description of factors related to knowledge, concerns, and confidence regarding the COVID-19 vaccination

Regarding COVID-19 vaccine knowledge and concerns, 31 (6.8%) participants felt adequately informed about the available vaccines and their usefulness. However, 109 (24.1%) expressed concerns about serious adverse effects (Table 2).

When the participants were asked if they had confidence in vaccines against COVID-19, 107 (23.6%) noted that they were confident of vaccine efficacy, and 78 (17.2%) believed in the benefit of all booster doses of the COVID-19 vaccine to ensure the effectiveness of the vaccine (Table 2).

Description of intention to get vaccinated against COVID-19

When the participants were asked about their future intentions regarding the COVID-19 vaccine, 51.2% (n=232) indicated that they did not plan to get vaccinated. However, nearly half of the participants would still consider getting vaccinated.

Associated factors with COVID-19 vaccine intention

In the bivariate analysis, several variables were found to be significantly associated with the intention to get the COVID-19 vaccine. These variables included having no income (52.1% versus 36%, p=0.008), perception of infection severity (72.1% versus 19.8%, p<0.001), personal history of COVID-19 infection (32.5% versus 52.4%, p=0.001), experiencing the death of a member of one’s social circle due to COVID-19 (63.8% versus 41.4%, p<0.001), belief in the vaccine’s efficacy (79.4% versus 39.3%, p<0.001), belief in the benefits of receiving all booster doses to ensure vaccine effectiveness (84.6% versus 41.3%, p<0.001), and concerns about serious adverse effects (15.6% versus 59.3%, p<0.001) (Table 1 and Table 2).

The logistic regression analysis assessed the factors that influence the intention to receive the COVID-19 vaccine, and several predictors were found to be significant (p<0.05). These predictors include having no income (AOR: 2.7, 95% confidence interval (CI): 1.9-5.1, p=0.003), believing that COVID-19 is a serious threat (AOR: 13.1, 95% CI: 7.6-22.3, p<0.001), not having had a COVID-19 infection (AOR: 3.3, 95% CI: 1.7-6.4, p=0.001), concerns about vaccine side effects (AOR: 8.6, 95% CI: 4.4-16.9, p<0.001), and belief in the benefits of booster doses (AOR: 5.5, 95% CI: 2.4-12.6, p<0.001) (Table 3).

**Table 3:** Associated factors with COVID-19 vaccine intention and the results of the logistic regression OR: odds ratio, CI: confidence interval, AOR: adjusted odds ratio Variables with a significance level of p<0.2 were included in the logistic regression model. A p-value of <0.05 was considered significant.

Variables	OR	95% CI	AOR	95% CI	p-value
Income					
No income	2	1.3-3.3	2.7	1.9-5.1	0.003
With income	1		1		
In your opinion, is the COVID-19 infection a serious infection?					
Yes	10.5	6.7-16.3	13.1	7.6-22.3	<0.001
No	1		1		
Have you already been infected with COVID-19?					
No	2.5	1.4-3.3	3.3	1.7-6.4	0.001
Yes	1		1		
Does the COVID-19 vaccine have any serious adverse effects?					
No	10	5.0-14.3	8.6	4.4-16.9	<0.001
Yes	1		1		
Do you believe in the benefit of all booster doses of the COVID-19 vaccine to ensure the effectiveness of the vaccine?					
Yes	7.8	4.1-14.9	5.5	2.4-12.6	<0.001
No	1		1		

## Discussion

To streamline the rollout of COVID-19 vaccines in Morocco, this study delves into the factors shaping vaccination intention among individuals who harbor hesitations about receiving the vaccine, despite its accessibility and cost-free nature. Notably, 48.8% of the participants indicated their intention to be vaccinated. However, this figure is lower than the global vaccination trend, which stands at 66.01% according to a comprehensive systematic review and meta-analysis of 63 surveys [16], and also lower than the 62.4% COVID-19 vaccine acceptance rate observed in Arab countries, as noted in another notable study [9].

Our results indicated a significant relationship between having no income and a high vaccine intention. This correlation could be because people without income may have limited access to health services and therefore perceive vaccination as a crucial preventive measure to avoid costly medical treatments in case of illness. However, this finding contrasts with other studies that found that individuals with higher monthly incomes were more likely to express willingness to receive the COVID-19 vaccine compared to those with lower incomes. This finding suggests that having a stable income allows individuals to prioritize their health, which includes getting vaccinated against diseases such as COVID-19 [9,17].

Consistent with previous research, our findings reveal a positive correlation between heightened personal perception of infection severity and the inclination to get vaccinated [18]. In other words, individuals who perceive COVID-19 as a significant threat prioritize protecting themselves and their social circles from the virus. Moreover, they may perceive vaccination as an efficient strategy to reduce the risk of infection and mitigate the severity of illness if they are exposed.

Research has further shown that an insufficient understanding of COVID-19, combined with a diminished perception of its severity, particularly among individuals with a personal history of COVID-19 infection, contributes to a low intention to get vaccinated. This finding aligns with our study, as the participants with a history of COVID-19 infection expressed a lower intention to vaccinate themselves compared to those without such a history [19].

Furthermore, concerns about vaccine side effects were found to be associated with vaccination intention. This finding aligns with previous studies that indicate a significant link between one’s willingness to accept a COVID-19 vaccine and concerns over minor adverse reactions [19-21]. This correlation highlights that the perception of safety significantly influences vaccine acceptance. Systematic reviews conducted in various countries, including Kuwait, Jordan, Italy, Russia, Poland, the United States, and France, consistently show a correlation between vaccination intention and increased confidence in vaccine safety and efficacy [11,17,22]. Global unease about vaccine confidence may be due to the novelty of these vaccines and the historical significance of the COVID-19 vaccine as the first to receive global emergency approval. This underscores the crucial role of educational policies in disseminating clear and credible information about vaccines and their effectiveness through all available communication channels. These efforts benefit not only individuals but also society at large, including children, communities, and families. The overarching goal is to strengthen public confidence in vaccination efforts.

Additionally, people who express skepticism about the need to be vaccinated with all booster doses to achieve maximum effectiveness often demonstrate a low vaccination intention, which indicates that they have underlying concerns, misconceptions, and barriers. This trend, consistent with the results of other studies [18, 23], underscores the significance of educational initiatives aimed at offering comprehensive information about vaccination in general, COVID-19 vaccination specifically, and the importance of booster doses. Building confidence in vaccines through evidence-based information and guidelines from professional associations, government authorities, and international institutions such as the WHO and Centers for Disease Control and Prevention (CDC) is crucial for fostering vaccine acceptance and ensuring the completion of recommended vaccine doses [24].

To the best of our knowledge, this study is the first to comprehensively examine the factors that influence vaccination intention in Morocco. By providing evidence-based insights, this research offers policymakers and health authorities critical information to address the challenges of vaccine intention within the context of COVID-19 vaccination. The strength of this study lies in its ability to identify key determinants of vaccine intention, which contributes valuable data to inform strategies aimed at increasing public engagement in mass vaccination campaigns. Despite its limitations, this research lays the foundation for future studies and initiatives focused on improving vaccine uptake and addressing hesitancy in the Moroccan population.

Limitations of the study

Our cross-sectional epidemiological analysis encounters challenges in establishing causality due to the inability to ascertain temporal relationships between variables. Moreover, the findings do not capture temporal changes or evaluate longitudinal effects accurately. However, future research should incorporate longitudinal designs and more diverse sampling methods to address these limitations and provide a more comprehensive understanding of the dynamic and multifaceted nature of vaccine acceptance in diverse populations.

## Conclusions

The engagement of communities in vaccination drives is a pivotal goal in attaining herd immunity. By identifying the key factors that influence vaccination intent, this study provides valuable insights that can strengthen public participation in vaccination campaigns and enhance adherence to mass vaccination programs, thereby improving the prevention of other infectious diseases.
